# Ultrasonic Vocalizations Emitted by Flying Squirrels

**DOI:** 10.1371/journal.pone.0073045

**Published:** 2013-08-29

**Authors:** Meghan N. Murrant, Jeff Bowman, Colin J. Garroway, Brian Prinzen, Heather Mayberry, Paul A. Faure

**Affiliations:** 1 Environmental and Life Sciences Graduate Program, Trent University, Peterborough, Ontario, Canada; 2 Wildlife Research and Development Section, Ontario Ministry of Natural Resources, Peterborough, Ontario, Canada; 3 Department of Psychology, Neuroscience & Behaviour, McMaster University, Hamilton, Ontario, Canada; Université de Bordeaux and Centre National de la Recherche Scientifique, France

## Abstract

Anecdotal reports of ultrasound use by flying squirrels have existed for decades, yet there has been little detailed analysis of their vocalizations. Here we demonstrate that two species of flying squirrel emit ultrasonic vocalizations. We recorded vocalizations from northern (*Glaucomys sabrinus)* and southern (*G. volans*) flying squirrels calling in both the laboratory and at a field site in central Ontario, Canada. We demonstrate that flying squirrels produce ultrasonic emissions through recorded bursts of broadband noise and time-frequency structured frequency modulated (FM) vocalizations, some of which were purely ultrasonic. Squirrels emitted three types of ultrasonic calls in laboratory recordings and one type in the field. The variety of signals that were recorded suggest that flying squirrels may use ultrasonic vocalizations to transfer information. Thus, vocalizations may be an important, although still poorly understood, aspect of flying squirrel social biology.

## Introduction

Exploration of the use of ultrasonic vocalizations by animals began in the 1930s when it was discovered that katydids emitted high frequency sounds above the range of human hearing [1]. Although the functions of ultrasonic vocalizations are well known for some animals, the use of ultrasound for signalling and communication is still unclear in many species. The purpose of our study was to document the use of ultrasonic vocalizations by two species of flying squirrel and describe the signals and the context in which they were recorded.

By definition, ultrasound is any signal with a frequency above 20 kHz, the upper frequency limit of human hearing [1]. As the frequency of a sound increases, its wavelength becomes shorter. Although ultrasonic frequencies reflect more easily from smaller objects they also are more readily absorbed by water molecules in the atmosphere and this results in a more rapid attenuation rate in air [2], [3]. As a result, animals that emit higher frequencies for the purpose of communication must either invest more energy into the vocalization, giving it relatively more power and allowing it to propagate farther in the air and be more readily detected by both intended and unintended recipients, or senders must remain closer to receivers [4].

Due to the shorter wavelengths of higher frequency sounds, organisms must possess adaptations for sending and receiving ultrasonic frequencies. For example, Masterton et al. [5] showed that head size directly correlates with the range of frequencies an organism can hear. Smaller heads are more efficient at blocking and detecting directionality of sounds with shorter wavelengths [6], [7]. For example, a small organism that listens to sound frequencies within the human audible range may possess a localization accuracy of 10° to 20°, but their accuracy substantially improves when they listen at higher frequencies [6].

The use of ultrasound for orientation, prey detection, and social interactions is well documented in echolocating bats [2], [8], [9]; however, many species of mammal use high frequency sounds for communication. For example, some studies have examined the role of ultrasound in alarm signalling [10]–[12], whereas others have documented how variation in ultrasonic vocalization structure can be used for additional functions [13]. A variety of studies have shown that mice, voles, and rats use ultrasound during courtship, mating, and child rearing [13]–[16]. Ultrasonic vocalizations are also used in non-reproductive social encounters [13], [14], [17], and are emitted by rodent pups in distress to attract parental attention and retrieval [18].

Since the early suggestion by Muul and Alley [19] that southern flying squirrels (*Glaucomys volans*) might echolocate, flying squirrels have been known to emit high frequency sounds, including ultrasound; but these vocalizations have only begun to be studied in greater detail. The use of ultrasound for navigation has never been demonstrated in flying squirrels, but these animals do appear to use high frequency sounds in a variety of contexts [19]–[21]. While undertaking field studies of flying squirrels, we have observed apparent signalling between squirrels using frequencies in both the audible and ultrasonic ranges. Because of their morphology, and life history and predator avoidance strategies, flying squirrels are good candidates for the use of ultrasound for acoustic communication. Due to their relatively small head and ear size [22], flying squirrels would gain sound localization benefits from the use of short, high frequency wavelengths. Flying squirrels are also nocturnal, so acoustic information may be an important supplement to visual signals. Furthermore, owls are among the main predators of flying squirrels [23], hence by using sound frequencies above the typical hearing range of owls, flying squirrels may benefit from increased predator avoidance. Owls typically hear in the range from about 500 Hz to 12 kHz, with the most sensitive region falling between 2 and 6 kHz [24]–[27]. Although some owl species, such as the barn owl (*Tyto alba*), can hear up to 13.8 kHz, this upper limit still falls well short of 20 kHz, which by definition is the start of the ultrasonic range [1], [24], [26]. Therefore, ultrasonic communication between flying squirrels should be largely undetectable by predatory owls.

Flying squirrels are social nesters [28], [29], and like many muroid species, they may use ultrasound for intraspecific communication within nest groups [30]. Flying squirrels forage alone for the majority of foraging events [31]; however, as social nesters, information transfer between nest mates outside of the nest may be necessary for the reassembly of social nesting groups, especially when the group switches to a new nest site [31]. Layne and Raymond [32] reported that members of social nesting groups would reassemble after being scattered while foraging, suggesting that communicative interactions between individuals could have facilitated the group's reassembly. Squirrels may also use high frequency vocalizations to share information while foraging within a group, such as the quality and location of food patches and as a warning of the presence of predators [33].

The purpose of our study was to record and analyze vocalizations produced by northern (*Glaucomys sabrinus*) and southern flying squirrels. Our goal was to record the types of vocalizations squirrels emitted, and document the extent of ultrasound use. While it is known that some flying squirrels emit high frequency sounds [19]–[21], ultrasonic vocalizations from *G. sabrinus* and *G. volans* have not been fully described, and little is known about the extent of use and functional significance of these vocalizations.

## Materials and Methods

### Ethics Statement

All procedures were approved by the Trent University Animal Care Committee (#08034) and the McMaster University Animal Research Ethics Board (AUP #08-07-34), and were in accordance with the guidelines of the Canadian Council on Animal Care.

### Laboratory Recordings

We live-trapped six flying squirrels (3 adult male and 2 adult female southern flying squirrels and 1 adult female northern flying squirrel) during autumn 2009 from a study site in the Kawartha Highlands (44.7°N, 78.3°W) using Tomahawk live traps (model 102; Tomahawk, WI, USA). Autumn is outside of the breeding season and so no individuals were reproductive. We do not have information about whether these individuals were related or whether they were part of the same social group. Evaluating the importance of social group membership and kinship for ultrasonic calling are beyond the scope of this work, however. Upon capture, squirrels were immediately transported to McMaster University (Hamilton, ON) where they were housed in a holding room in individual plastic animal cages (35×28×24 cm; l×w×h). Each cage had fresh bedding material consisting of wood chips and natural cotton. These cages were used for housing and during recording sessions. With no acclimatization period squirrels were kept indoors on a 12:12 light:dark photoperiod (standardized to Toronto, ON). Fresh water and food (apple slices, pine nuts, and peanut butter) were provided *ad libitum*. After our recordings were completed, all squirrels were released at their original capture location.

Recording sessions lasted an average of 3.5 hrs per night, beginning at approximately 1800 hrs as squirrels became active. Over an 8 day period we collected 29.33 hrs of recordings. The recording room measured 4.85×3.25×3.32 m and was lined with sound attenuating foam (Sonex® Classic; Pinta Acoustic, USA). Sounds were recorded with two CM16 condenser microphones (Avisoft Bioacoustics, Berlin Germany) mounted on miniature tripods, each placed 15 to 20 cm from the centre of the cage. The output from each microphone was fed to a 4-channel digitizer (gain 8, sampling rate 250 kHz, 16 bit amplitude resolution, UltraSoundGate 416-200; Avisoft Bioacoustics) connected to the USB port of a laptop running Avisoft Recorder software.

Squirrels were brought into the recording room, each in separate cages. We had separate recording periods for individual squirrels alone in the room as well as for pairs of squirrels housed in separate cages. Pairs consisted of squirrels of the same species (conspecifics) and a mix of northern and southern flying squirrels (heterospecifics). During recording sessions with conspecifics, squirrels were kept in separate cages with approximately 75 cm between each cage. Squirrels were allowed to roam their cages during recording sessions and such movements created non-vocal sounds that could be detected. We classified five groups of non-vocal sounds – eating, drinking, bar chewing, cage exploration, and wheel running – and eliminated them from our final analysis. A sample of the sound created from each action was recorded during visual confirmation of the non-vocal behaviour; the animals were monitored via infrared video in the darkened sound room.

Recorded sounds were analyzed with the Sound Analysis and Synthesis Laboratory Professional software (Avisoft SASlab Pro). Vocalizations in the recorded files were labelled (amplitude threshold criterion 5%, hold time 20 ms) and a spectrogram was generated to facilitate the measurement of temporal (resolution  = 0.016 ms) and spectral parameters (resolution  = 976 Hz). A higher temporal resolution was chosen at the sacrifice of lower frequency resolution because the observed sounds had short durations but large bandwidths. Temporal measures included call duration, defined as the time between the onset and offset of the vocalization, and interpulse interval (IPI), defined as the time between the onset of successive vocalizations emitted in sequence. Spectral parameters included the minimum and maximum frequency, and the frequency of maximum energy (i.e., peak spectral frequency).

### Field Recordings

A field study was undertaken in central Ontario at the Trent University James McLean Oliver Research Centre (44.6°N, 78.5°W). The site was a mature 38-ha hardwood woodlot with an overstory dominated by *Acer saccharum*, but also including *Quercus rubra*, *Betula papyrifera*, *Fagus grandifolia*, *Fraxinus americana*, *Pinus strobus*, *Populus tremuloides*, *Prunus serotina*, *Tilia americana*, and *Thuja occidentalis*.

The study area contained a population of *G. volans* that have been under investigation for several years [28], [34], [35]. As a result of consistent live trapping at this site, the entire population of squirrels was marked with passive integrated transponders (PIT tags, Eidap Inc., Sherwood Park, AB). A select group of squirrels were fitted with radio collars (1.8 g model BD-2C, Holohil Inc., Carp, ON) and this allowed us to track them to their nest locations [28]. We monitored nest sites with remote, data logging PIT readers, hence we were able to record and identify each squirrel occupying a nest at any given time.

At select nest cavities in the summer of 2011, a single batcorder (ecoObs, Nürnberg Germany) was placed 20 cm from the nest entrance (microphone 0° incidence) to record squirrel vocalizations. The batcorder was activated just prior to dusk and was set to record for 4 hours. The batcorder was set with a critical frequency of 14 kHz, a quality of 20, and a post trigger of 400 ms. The recorder was set to automatically activate and record detected sounds >14 kHz. A trail camera was deployed at each nest cavity to capture movements around the nest cavity during the period of recording. The batcorder was sequentially placed at three separate nest cavities and remained there between 2 to 4 nights.

### Data Archiving

Characteristic sound recordings of each type of flying squirrel vocalization are available as wav files at datadryad.org, an open-access data repository (doi:10.5061/dryad.s44gk).

## Results

### Laboratory Recordings

The majority of sounds recorded in the lab were non-vocal, caused by behaviours such as eating, drinking, cage climbing, and wheel running; however, we recorded three types of sounds that were determined to be squirrel vocalizations.

**Figure 1:**
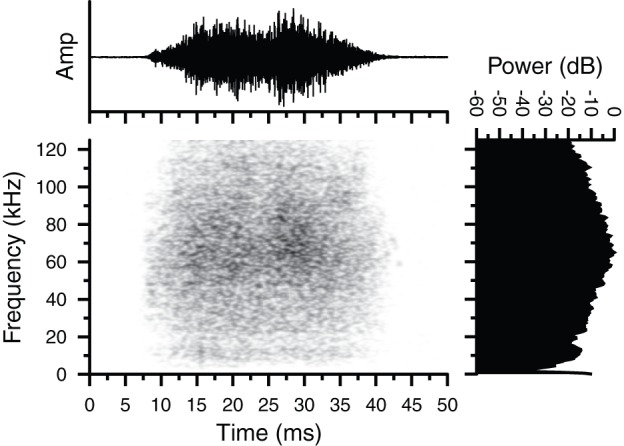
Oscillogram, spectrogram and magnitude spectrum of a Type 1 broadband noise burst. The vocalization was emitted by a captive female *Glaucomys volans* calling in a room lined with sound attenuating foam at McMaster University. Call duration  = 31.29 ms; peak spectral frequency  = 65.0 kHz.

The first type of vocalization that we characterized, which we refer to as Type 1, was a broadband noise burst with no time-frequency structure that had a duration of 20–40 ms and peak frequencies regularly exceeding 60 kHz (Fig. 1). These sounds were recorded in a variety of situations, including: throughout cage exploration, between feeding events, and while climbing within the cage. Broadband noise bursts were recorded both from isolated individuals and in the presence of conspecifics, and were produced by both male and female *G. volans* and a female *G. sabrinus*. Type 1 broadband noise bursts made up 95% of all vocalizations recorded in the lab (Type 1 n = 73; Type 2 n = 2; Type 3 n = 2).

**Figure 2:**
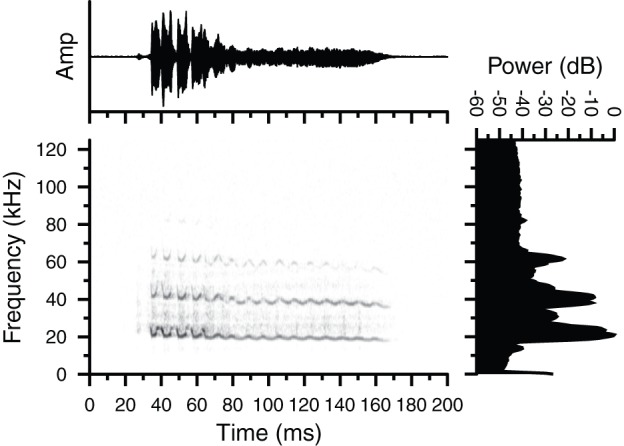
Oscillogram, spectrogram and magnitude spectrum of a Type 2 vocalization emitted by a male *Glaucomys volans* in proximity to a female conspecific. Shown is one of four calls emitted in the sequence. Both animals were house separately in a room lined with sound attenuating foam at McMaster University. Note the prominent frequency (FM) and amplitude modulations (AM) in the signal, and the clear presence of harmonics. Call duration  = 135 ms.

Type 2 vocalizations consisted of a long duration signal with large amplitude modulation (AM) at the beginning of the call and a sinusoidal frequency modulation (SFM) structure. For the Type 2 call shown, the SFM changes within the fundamental acoustic element initially oscillated between about 27 and 19 kHz but eventually narrowed in SFM bandwidth (Fig. 2). These sounds were produced once in a set of three vocalizations by a female *G. volans* in isolation, and once in a set of four vocalizations by a male *G. volans* in the presence of a female conspecific (Table 1). Sounds from the set of three vocalizations were composed of a fundamental acoustic element with either zero or one overtone (harmonic) at twice the frequency of the fundamental element, whereas sounds from the set of four vocalizations had two to three overtones, each at an integer multiple of the fundamental element (Fig. 2).

**Table 1:** Acoustic parameters of two sets of sinusoidal frequency modulated (SFM) vocalizations (Type 2) produced by a female (set of 3) and male (set of 4) southern flying squirrel (*Glaucomys volans*) recorded in a room lined with sound attenuating foam at McMaster University.

	Duration (ms)	IPI (ms)	Min (kHz)	Max (kHz)
Set of 3				
1	44	-	20.9	25.8
2	109	231	19.5	26.3
3	65	290	19.5	26.8
Set of 4				
1	105	-	18.7	25.1
2	135	285	20.8	26.3
3	79	285	18.5	25.8
4	60	250	19.0	26.1

Parameters included call duration, interpulse interval (IPI), minimum and maximum frequency.

**Figure 3:**
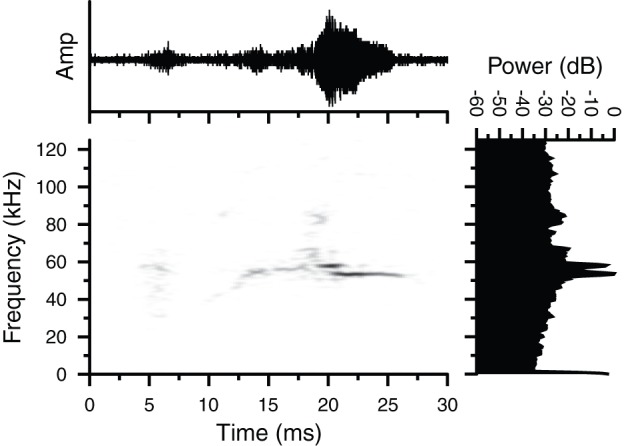
Oscillogram, spectrogram and magnitude spectrum of a Type 3 vocalization. The FM signal was emitted by a male *Glaucomys volans* in proximity to a female conspecific. Both animals were house separately in a room lined with sound attenuating foam at McMaster University. The signal consists of two-note vocalization with quasi-constant frequency (pure-tone) signals comprising each note. Call duration  = 14.5 ms.

We recorded two examples of Type 3 vocalizations and both were emitted by a male *G. volans* in the presence of a female conspecific. The total duration of the Type 3 sound shown in Figure 3 was 14.5 ms and the call consisted of a two-note signal. The first note was an upward frequency modulated (FM) sweep increasing in amplitude from 55.6 to 58.5 kHz over a duration of 10 ms. The second note was a 7.5 ms narrowband, quasi-constant frequency (CF) tone of 53.7 kHz that overlapped the first note for 3 ms and continued for 4.5 ms (Fig. 3).

### Field Recordings

**Figure 4:**
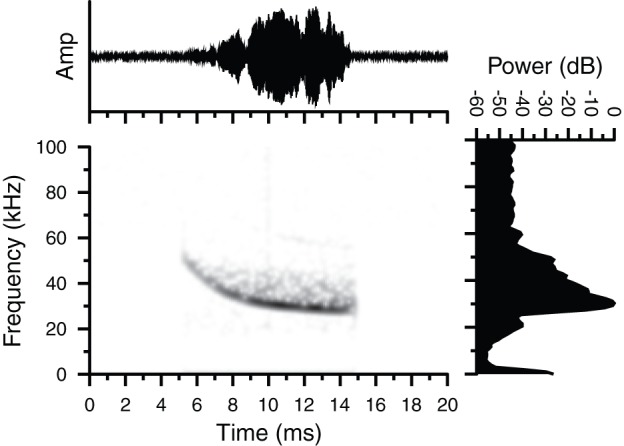
Oscillogram, spectrogram and magnitude spectrum of a Type 4 vocalization. The call was recorded in the field from a male *Glaucomys volans* calling from an occupied nest cavity. Note the hyperbolic frequency modulated (FM) structure. Call duration  = 10 ms.

While handling captured squirrels in the field, we recorded Type 1 broadband noise bursts from both *G. volans* and *G. sabrinus* on several occasions and these vocalizations had similar temporal and spectral parameters to the broadband noise burst we recorded in the laboratory (Fig. 1). We also recorded a fourth type of FM vocalization (Type 4) at an active *G. volans* nest cavity. This broadband signal was a purely ultrasonic hyperbolic downward FM sweep that began at approximately 51 kHz and swept down to 28 kHz over a duration of 10 ms (Fig. 4). Type 4 vocalizations recorded in the field were emitted in sets of 2, 3, or 5, and all came from a single nest with 3 *G. volans*. PIT-tag records indicated that the calls corresponded to the arrival of one male squirrel at the nest with two conspecifics already inside.

## Discussion

We have confirmed that flying squirrels emit ultrasonic vocalizations. Furthermore, we recorded at least four distinct call types, including an unstructured broadband noise burst that spanned both audible and ultrasonic frequencies (Fig. 1), and purely ultrasonic vocalizations with clear time-frequency structure (Figs. 2–4). The presence of highly structured sounds suggests that the vocalizations may serve as a means of information transfer and are used for communication. Flying squirrels are social animals and the fact that they emit a variety of vocalizations strongly suggests that these signals reflect an important aspect of their social behaviour.

Type 1 broadband noise burst vocalizations were emitted by both species of flying squirrel in all situations in the lab (Fig. 1). Squirrels emitted Type 1 vocalizations alone and in the presence of conspecifics, while exploring their cages, while stationary in their nests, and while being handled. Because Type 1 vocalizations contained no clear time-frequency structure and were produced in multiple situations, it is difficult to speculate on what information content the signals may carry. It is possible that Type 1 signals simply serve as a contact call between conspecifics. Owing to their large bandwidth, Type 1 calls are predicted to be easy to detect and localize by conspecifics [6]. Sounds that span a greater range of frequencies provide more “listening channels” for extracting interaural time and level difference cues important for sound localization [36]. Broadband noise bursts have the potential to be more detectable to a large range of listeners, including potential predators. Such calls would therefore appear to be disadvantageous; however, there are also benefits to emitting broadband sounds. If Type 1 calls are emitted by squirrels to maintain acoustic contact with conspecifics while exploring the environment, then incorporating a wider range of frequencies enhances the probability of signal detection [37]. By extending the signal into the audible frequency range, the low frequency components of the call will propagate farther and diffract around obstacles in the environment and thus reach a wider audience compared to higher frequency sounds with shorter wavelengths [1], [6]. Therefore, incorporating both audible and ultrasonic frequencies into Type 1 vocalizations enables this signal to be heard over a large operating area and still be accurately localized.

Flying squirrels emitted three types of structured FM vocalizations in both lab and field settings. Type 2 vocalizations contained prominent AM and sinusoidal FM components. Type 3 vocalizations contained a lower amplitude upward FM with overlapping CF components at the end of the call. Type 4 vocalizations were hyperbolic downward FM sweeps not unlike the biosonar calls emitted by many species of laryngeally echolocating bats [38]. Although we are confident that the Type 4 vocalizations originated from a tree cavity containing flying squirrels, the uncontrolled nature of recording sounds in the field suggests that we remain cautious about fully accepting that squirrels were the source of the Type 4 calls until we can carry out further controlled recordings.

Frequency modulated vocalizations are also emitted by a variety other mammals in different contexts. Albino male rats emit FM signals after tail stimulation [39]. Marmots (*Marmota* spp.) use both upward and downward FM in their alarm calls [40]. Free living mice (*Peromyscus* spp.) produce a range of ultrasonic vocalizations that likely serve communication functions [30]. A key feature of these FM calls is their time-frequency structure, which increases information content of the signal. Mammalian FM vocalizations can provide very specific information to listeners, such as the location and behaviour of conspecifics and predators, and the identity of individuals [7], [41]–[43].

Some of the time-frequency structured FM vocalizations emitted by *G. volans* were purely ultrasonic whereas in others the end of the downward sweep grazed into the audible frequency range. If a FM call incorporates both ultrasonic and audible frequencies, it might serve a similar function to the broadband Type 1 calls if the intent is to transmit information as far as possible while also being localizable to conspecifics. This suggests the hypothesis that purely ultrasonic vocalizations are most likely to be emitted when conspecifics are in close proximity. When squirrels forage together they may opt to communicate with entirely ultrasonic signals in order to share food quality information while simultaneously decreasing the probability of being detected by avian predators. This appears to be the case for Richardson's ground squirrels (*Urocitellus richardsonii*), who have adopted a strategy of alternating between pure ultrasonic, audible, and ‘mixed’ alarm call signalling [11]–[12]. Mixed alarm calls were emitted by ground squirrels when predators were at a distance so neither the sonic nor ultrasonic components of the signal could easily be detected by the predator but would be audible to nearby conspecifics. When predators were detected at intermediate distances, ground squirrels switch to emitting purely ultrasonic signals that would be less likely to reach the predator but would still be heard by nearby conspecifics and thereby elicit a response. When predators were detected at close range, ground squirrels switch to an alarm call in the sonic frequency range, giving the call a greater propagation distance and increasing its warning impact [11], [12].

Flying squirrels may also use ultrasound for communication at the nest, either in maternal-offspring groups as in other rodents [18], [44], or during the winter when they form larger, non-kin, social nesting groups. We speculate that socially-nesting squirrels may use ultrasonic and mixed frequency vocalizations to aid in regrouping at a nest cavity after a bout of nocturnal foraging. Once squirrels form a nest group, the cohesiveness of the group is maintained and individuals reside within the same group for the remainder of the season [28], [32]. Flying squirrels use the same nest cavity for the majority of a season, although occasionally they switch nesting locations suggesting a need for information transfer between group members. A question that remains is: how do squirrels from the same non-kin social group coordinate their nocturnal movements so that they all end up at the new nest site? We speculate that flying squirrels rely on ultrasonic vocalizations while regrouping in order to avoid detection from predators.

Recently, Gilley [21] described vocalizations by both northern and southern flying squirrels recorded in a lab setting. Gilley characterized 3 call types for each species, and it appears that only one of these is similar to a call we recorded. Our Type 2 vocalization is similar to a *G. volans* call that Gilley [21] referred to as a trill. Both calls are frequency modulated and of similar duration, and both calls were emitted in sets of 3–5 syllables. Gilley [21] did not report squirrel calls similar to our Types 1, 3, or 4. Thus, the vocal repertoire of southern flying squirrels appears to contain at least 6 different call types, although it is possible that some of this variation may be due to regional differences.

Muul and Alley [19] suggested that flying squirrels could use ultrasound to aid in navigating while gliding, similar to echolocation by bats. Although flying squirrels have large ebony eyes, and undoubtedly use visual cues during glides, their nocturnal habits and heavily forested environment suggest that additional navigational cues – such as listening to reflections of their own voices – may also be helpful. Chattin [20] tried to demonstrate that *G. volans* used ultrasound in flight to help them detect suitable landing locations, but was unable to detect vocalizations during the glides of his captive animals. We also were unable to detect sounds of squirrels while in flight, although this was not our major objective. We are unaware of any data that directly support the hypothesis that flying squirrels use ultrasound to aid in navigation while gliding, and suggest that this hypothesis remains to be adequately tested. An experimental assessment of captive squirrels in a sound lab outfitted for gliding would be ideal to address this hypothesis.

Behavioural observations of flying squirrels in the lab during our recording sessions suggests that they react to their own high frequency vocalizations, and are therefore able to hear ultrasound. This observation is consistent with the work of Chattin [20], who was able to demonstrate that flying squirrels reacted to sound frequencies as high as 75 kHz. We tried to extend this work by conducting a playback experiment at our field site using a Type 2 vocalization recorded in the lab. Broadcasts of this ultrasonic vocalization alone, as well as in combination with a dummy flying squirrel, failed to elicit observable responses from the squirrels in our study population.

In addition to confirming that flying squirrels emit ultrasonic vocalizations, our study provides a starting point for future studies to examine the function of these vocal signals. We recorded a variety of call types in both the audible and ultrasonic ranges that were emitted by flying squirrels in both the lab and the field. Some of these signals possessed clear time-frequency structure, suggesting they may serve a communicative function. Additional studies are needed to better appreciate the sensory capabilities of flying squirrels and the behavioural contexts under which they emit vocalizations. Future studies should include behavioural (psychophysical) experiments that manipulate visual and acoustic cues in different sensory tasks, and neurophysiological studies of auditory function. We suspect that ultrasonic vocalizations are an important aspect of flying squirrel ecology that has been heretofore under appreciated.
